# Differential Macrophage Responses in Affective Versus Non-Affective First-Episode Psychosis Patients

**DOI:** 10.3389/fncel.2021.583351

**Published:** 2021-02-24

**Authors:** Heather K. Hughes, Emily Mills-Ko, Houa Yang, Tyler A. Lesh, Cameron S. Carter, Paul Ashwood

**Affiliations:** ^1^Department of Medical Microbiology and Immunology, University of California at Davis, Davis, CA, United States; ^2^MIND Institute, University of California at Davis, Sacramento, CA, United States; ^3^Department of Psychiatry and Behavioral Sciences, University of California at Davis, Sacramento, CA, United States

**Keywords:** psychosis, schizophrenia, bipolar disorder, major depressive disorder, affective, immune, macrophage, inflammation

## Abstract

Increased innate immune activation and inflammation are common findings in psychotic and affective (mood) disorders such as schizophrenia (SCZ), bipolar disorder (BD), and major depressive disorder (MDD), including increased numbers and activation of monocytes and macrophages. These findings often differ depending on the disorder, for example, we previously found increases in circulating inflammatory cytokines associated with monocytes and macrophages in SCZ, while BD had increases in anti-inflammatory cytokines. Despite these differences, few studies have specifically compared immune dysfunction in affective versus non-affective psychotic disorders and none have compared functional monocyte responses across these disorders. To address this, we recruited 25 first episode psychosis (FEP) patients and 23 healthy controls (HC). FEP patients were further grouped based on the presence (AFF) or absence (NON) of mood disorder. We isolated peripheral blood mononuclear cells and cultured them for 1 week with M-CSF to obtain monocyte-derived macrophages. These cells were then stimulated for 24 h to skew them to inflammatory and alternative phenotypes, in order to identify differences in these responses. Following stimulation with LPS and LPS plus IFNγ, we found that macrophages from the NON-group had diminished inflammatory responses compared to both HC and AFF groups. Interestingly, when skewing macrophages to an alternative phenotype using LPS plus IL-4, the AFF macrophages increased production of inflammatory cytokines. Receiver operating curve analysis showed predictive power of inflammatory cytokine concentrations after LPS stimulation in the AFF group versus NON-group. Our results suggest dysfunctional monocyte responses in both affective and non-affective psychotic disorder, with varying types of immune dysfunction depending on the presence or absence of a mood component.

## Introduction

Psychotic and affective (mood) disorders, including schizophrenia (SCZ), bipolar disorder (BD), and major depressive disorder (MDD) cause significant disability and are a major public health concern (Reddy, 2010; Ferrari et al., 2012). The burden of these neuropsychiatric disorders are likely under-estimated, despite their appearance within the top 20 causes of global burden of disease in 2013 (Vigo et al., 2016). The exact cause of these disorders is yet unknown, however findings from genetic and epidemiological studies indicate that the interplay of environmental and genetic components contribute to the development and severity of neuropsychiatric disorders (Uher and Zwicker, 2017). Inflammation and immune dysfunction are common findings in these disorders, with activation of the immune system and increased inflammatory mediators proposed as possible etiological mechanisms (reviewed in Bauer and Teixeira, 2019). Chronic infections, inflammatory conditions and autoimmunity are considered risk factors for psychotic and affective disorders (Benros et al., 2013, 2014; Rosenblat and McIntyre, 2015). Several meta-analyses have consistently found elevations of peripheral and central inflammatory cytokines and chemokines across these disorders (Dowlati et al., 2010; Modabbernia et al., 2013; Munkholm et al., 2013; Goldsmith et al., 2016; Pillinger et al., 2018; Wang and Miller, 2018). As the majority of these cytokines and chemokines are produced predominantly by cells of the innate immune system, an overarching hypotheses that connects the immunological dysfunction of these disorders is aberrant activation of innate immunity. Originally described for SCZ as a “chronically activated inflammatory response system” (Smith and Maes, 1995), this hypothesis proposes that alterations in the numbers and activation states of peripheral monocytes and macrophages, and microglia in the brain, lead to the increased inflammatory mediators commonly seen in psychotic and affective disorders and may be contributing to their etiology by altering brain function (Drexhage et al., 2010a; Beumer et al., 2012).

Monocytes and macrophages, and their counterpart the microglia in the brain, are myeloid cells of the innate immune system. As first responders in the immune response, their activity during activation is critical for orchestrating inflammatory or alternative/anti-inflammatory responses, as they produce mediators that help initiate and sustain adaptive responses (Geissmann et al., 2010). Circulating monocytes are present in humans as a heterogenous population, the majority of which are classical CD14^++^CD16^–^ monocytes, with intermediate CD14^hi^CD16^lo^ and alternative CD14^+^CD16^++^ monocytes making up 10% (Italiani and Boraschi, 2014). When recruited to tissues during immune activation, these cells differentiate into macrophages, which have been recently highlighted in their ability to polarize pro-inflammatory versus anti-inflammatory responses under various circumstances. Two states exist in the simplistic model of macrophage polarization: “M1” are the classically activated inflammatory macrophages, producing pro-inflammatory cytokines such as interleukin (IL-1)-β, IL-6 and tumor necrosis factor (TNF)-α to increase the inflammatory response. “M2” macrophages are alternatively activated anti-inflammatory cells, involved in wound healing and restoring homeostasis after inflammatory events by secreting anti-inflammatory cytokines and growth factors that help reduce and prevent further damage from inflammatory immune responses and promote recovery. In reality, these cells polarize along a spectrum, not strictly M1/M2 as these represent extremes of the spectrum and M2 has further subcategories that vary in function depending on the type of stimuli. However, macrophages can be polarized *in vitro* by stimulating with interferon (IFN)-γ or IL-4 to drive them toward M1 versus M2 phenotypes, respectively (Italiani and Boraschi, 2014).

Alterations in numbers and function of monocytes and macrophages have been repeatedly identified in these disorders, with varying degrees of dysfunction depending on the disorder(s) studied. Increases in circulating monocytes and cerebrospinal fluid monocytes were found repeatedly in SCZ and BD (Zorrilla et al., 1996; Rothermundt et al., 1998; Nikkila et al., 1999; Barbosa et al., 2014). When comparing gene expression profiles of monocytes from SCZ and BD patients, two clusters of highly correlated genes were identified in BD, one involving inflammatory mediators and the other involving motility and adhesion. However, in SCZ only the pro-inflammatory cluster was seen, and the motility/adhesion cluster was downregulated (Drexhage et al., 2010b). Elevated inflammatory gene expression in the monocytes of BD patients during mood episodes, compared to during the euthymic state was also found (Becking et al., 2015). More recently, an increased M1/decreased M2 gene expression signature was identified in peripheral blood mononuclear cells (PBMC) from BD patients but not from patients with SCZ, including increased *il-6* and *ccl3* (encodes for macrophage inflammatory protein [MIP]-1α) (Brambilla et al., 2014). Other studies have identified increased responses of stimulated monocytes from mood disorder patients. Knijff et al. (2007) found significantly altered pro-inflammatory responses in monocytes from BD patients compared to controls after lipopolysaccharide (LPS) stimulation, including increased IL-6. Conversely, when monocyte-derived macrophages from SCZ patients were stimulated with LPS or IL-4, no differences in functional responses at the gene-expression level were found, indicating a normal response to stimulation in SCZ macrophages (Ormel et al., 2017).

Additional support for the hypothesis that myeloid cells are dysfunctional in neuropsychiatric disorders are the repeated findings of increased inflammatory cytokines associated with these cells in SCZ, BD, and MDD. For example a meta-analysis of 30 BD cytokine studies showed significant increases in inflammatory cytokines and soluble receptors with variations in expression depending on manic or depressive episode (Modabbernia et al., 2013). IL-6 was elevated in acutely ill patients when comparing across studies of SCZ, BD, and MDD, which decreased in SCZ and MDD but not bipolar mania after treatment (Goldsmith et al., 2016). Elevated IL-6 mRNA and protein are proposed biomarkers for SCZ and MDD, respectively (Mössner et al., 2007; Chase et al., 2016). These cytokines typically associate with innate inflammation, supporting myeloid dysfunction in these disorders.

Taken together, findings of increased inflammatory cytokines and myeloid cell activation suggest that there is a relationship between innate immune activation and psychotic and affective disorders. Given this, we sought to identify differences in activation of monocyte-derived macrophages from first episode psychosis (FEP) patients with and without mood disorders. We also sought to compare macrophage responses to those of healthy controls. By stimulating macrophages toward M1 and M2 phenotypes, we aimed to identify variations in production of inflammatory cytokines and chemokines associated with monocytes, macrophages, and microglia to support the hypothesis of innate immune cell activation and dysfunction in psychotic and affective disorders, and to potentially address discrepancies seen in previous studies.

## Materials and Methods

### Study Participants

We recruited a total of 48 participants between the ages of 14 and 37 years old, including 23 healthy controls and 25 first episode patients with a psychotic disorder. Diagnoses included 10 SCZ, 1 psychosis not otherwise specified, 8 BD, and 6 MDD. Psychosis participants were outpatients within one year of onset of symptoms. Trained clinicians assessed patients using the Structured Clinical Interview for the DSM-IV-TR (SCID)-I/P (First et al., 2002) and symptoms were rated on several scales including the Scale for the Assessment of Negative Symptoms (SANS) (Andreasen, 1983), Scale for the Assessment of Positive Symptoms (SAPS) (Andreasen, 1984), and Brief Psychiatric Rating Scale (BPRS) (Lukoff et al., 1986). The majority of patients in both groups were on monotherapy antipsychotic medication. Exceptions included two individuals in the AFF group that were taking more than one antipsychotic, five unmedicated participants the AFF group and two unmedicated participants in the NON-group. One patient in the AFF group was also taking antidepressants in addition to an atypical antipsychotic. When this patient was omitted from the analyses, it did not alter outcome therefore the patient was included. Antipsychotic dosage was converted to chlorpromazine (CPZ) equivalent dose to assess relative antipsychotic potencies. Participants were excluded for positive urine toxicology at the time of testing, for alcohol or drug abuse/dependence within three months of assessment, and/or who had a Wechsler Abbreviated Scale of Intelligence (WASI) IQ score that was below 70. In addition, healthy controls (HC) were excluded for presence of any Axis I or Axis II disorder, or psychotic disorder within first degree family members. Diagnoses was later confirmed at a 12-month assessment. The University of California, Davis Institutional Review Board approved this study.

### Cell Isolation

Peripheral blood was collected from each participant in an acid-citrate-dextrose Vacutainer tube (BD Biosciences, San Jose, CA, United States) and processed within 12 h of collection. The blood was centrifuged for 10 min at 2,100 rpm, then plasma was collected and stored at −80°C. The remaining blood was diluted 1:2 with Hanks Balanced Salt Solution (HBSS; Gibco, Gaithersburg, MD, United States) then carefully layered over a Ficoll-Paque gradient (Pharmacia Biotech, Piscataway, NJ, United States) and centrifuged at 1,700 rpm for 30 min at room temperature. PBMC were collected from the interface layer and washed twice with HBSS. Cell viability was determined by trypan blue exclusion. The cells were resuspended at a final concentration of 1 × 10^6^ cells/mL in tissue culture medium consisting of RPMI-1640 (Gibco) supplemented with 10% low endotoxin heat inactivated fetal bovine serum (Omega Scientific, Tarzana, CA, United States), 100 IU/mL penicillin, and 100 IU/mL streptomycin (Sigma, St Louis, MO, United States).

### Macrophage Growth and Stimulation

Freshly collected PBMC were plated in RPMI with 100 ng/mL recombinant human macrophage colony stimulating factor (rh-M-CSF, R&D Systems; Minneapolis, MN, United States) in Corning Ultra-Low Binding 100 × 20 mm dishes at an approximate density of 10 million cells in 10 mL volume. Media and rh-M-CSF were replenished after three days, and cultured for a total of 1 week, at which time plates were washed with HBSS to remove all but adherent macrophages. Adherent cells were then incubated for 5 min on ice with Cell Stripper (Corning, Manassas, VA, United States) and then removed by vigorously washing plates. Macrophages were plated at 2.5 × 10^4^ cells/well and cultured at 37°C with 5% CO_2_. The cells were stimulated in duplicate either with RPMI alone, or RPMI plus 10 ng/mL LPS (Sigma-Aldrich; St. Louis, MO, United States), or 50 ng/ml IFNγ (R&D Systems) and 10 ng/mL LPS, or 40 ng/mL IL-4 (R&D Systems) and 10 ng/mL LPS. After 24 h, plates were centrifuged at 2,100 rpm for 10 min and supernatants were collected and stored at −80°C until analysis.

### Cytokine and Chemokine Analysis

To assess macrophage inflammatory responses, supernatants from stimulated macrophage cultures were quantified for IL-1β, IL-6, IL-12p40, IL-12p70, and TNFα. Chemokine production was assessed by measuring monocyte chemoattractant protein (MCP)-1 (C–C chemokine ligand [CCL]2), MIP-1α (CCL3), and MIP-1β (CCL4). Granulocyte-macrophage colony-stimulating factor (GM-CSF) and IL-10 were measured to identify production of growth and repair mediators. These measurements were made using a high sensitivity Multi-Plex bead set (Millipore, Saint Charles, MO, United States). Samples were run in duplicate. Based on manufacturer’s recommendations, supernatants were incubated with antibody-coupled fluorescent beads overnight at 4°C in a humidified box. Plates were then washed. Biotinylated detection antibodies were then added to each well and incubated at room temperature for 1 h, followed by streptavidin–phycoerythrin and an additional 30 min incubation. The samples were analyzed using a flow-based Luminex^TM^ 100 suspension array system (Bio-Plex 200; Bio-Rad Laboratories, Inc.). Unknown sample cytokine concentrations were calculated by Bio-Plex Manager software using a standard curve derived from the known reference cytokine standards provided in each kit. The sensitivity of this assay allowed the detection of cytokine concentrations with the following limits of detection: IL-12(p40) (12.3 pg/mL), IL-12(p70) (0.9 pg/mL), IL-1β (0.7 pg/mL), IL-6 (0.4 pg/mL, TNFα (0.2 pg/mL), MCP-1 (1.2 pg/mL), MIP-1α (6.6 pg/mL), MIP-1β (3.2 pg/mL), GM-CSF (2.3 pg/mL), and IL-10 (0.3 pg/mL). Values below the limit of detection (LOD) were replaced with one-half the LOD.

### Statistical Analysis

Shapiro–Wilk test determined that the majority of the cytokine data were not normally distributed. Outliers were removed using ROUT with a coefficient Q set to 1%. Kruskal–Wallis tests were used to analyze differences across the three groups and Mann–Whitney *U* tests were then used for pair-wise analyses, with Holm–Sidak method to correct for multiple comparisons. To evaluate whether an inflammatory macrophage profile may discern between individuals with affective and non-affective psychosis, we ran binary logistic regression on cytokine levels that were log2 transformed to improve the goodness of fit as measured by the Hosmer–Lemeshow statistic, where a non-significant probability value indicates good fit (Grund and Sabin, 2010). Predictive power was evaluated by receiver operating characteristics analysis. To correct for the potential impact of antipsychotic medication on cytokine production, supplementary analyses were performed comparing cytokine concentrations between the AFF and NON-group by conducting both Mann–Whitney *U* tests in medicated versus non-medicated patients, and one-way analysis of covariance (ANCOVA). In order to meet assumptions required for ANCOVA, cytokine concentrations were rank transformed across groups, then separate ANCOVAs were performed for each cytokine including antipsychotic dose as a covariate, based on procedures described previously (Lesh et al., 2018). Statistical analyses were carried out using analyses software SPSS Statistics Version 26 (IBM, Armonk, NY, United States) and GraphPad Prism v7.0e (GraphPad Software, San Diego, CA, United States). A two-tailed alpha of *p* < 0.05 was considering statistically significant.

## Results

### Demographics and Clinical Characteristics

Table 1 summarizes study participant demographics and clinical scores. We recruited 48 participants which included 25 FEP participants and 23 healthy control (HC) subjects. FEP participants were further categorized based on the presence or lack of a primary affective disorder into affective (AFF), and non-affective (NON) groups. All participants were between the ages of 14 and 37, and there were no statistically significant differences in age between groups. The NON-group was male-skewed, however significant differences across groups were not evident based on Fisher’s exact test. Parent education level was similar across all three groups, however participant education level was significantly lower in both the AFF (*p* = 0.0121) and NON (*p* = 0.0016) groups compared to the HC group. BPRS and SAPS scores between both AFF and NON-populations did not differ, however the AFF group scored significantly lower on SANS (*p* = 0.0435) compared to the NON-group. WASI scores did not differ across groups. No significant differences were seen in dose of antipsychotic medication using CPZ equivalent doses for estimation, and cytokine concentrations were not significantly different in medicated versus non-medicated patients, based on Mann–Whitney U tests of significant cytokines in AFF and NON-groups (all *p* > 0.05). Supplementary ANCOVA analyses of rank-transformed significant cytokines with antipsychotic dosage equivalent (CPZ) as a covariate had no impact on the results (Supplementary Table S1).

**TABLE 1:** Demographic characteristics and clinical scores.

				*P* values		
	CTL (*n* = 23)	AFF (*n* = 14)	NON (*n* = 11)	CTL vs AFF	AFF vs NON	CTL vs NON
Age: median	22.4	19.4	19.7	0.246	>0.9999	0.4433
Range [minimum, maximum]	[14.5, 36.8]	[15.7, 24.2]	[15.5, 35.2]			
IQR	20.2–24.4	17.1-23.4	16.3-24.0			
Gender (%male/female)	65/35	57/43	91/9	0.7321	0.0900	0.2137
Subject education: median (IQR)	14 (14–16)	12 (10–14)	11 (9–13)	*0.0121*	>0.9999	*0.0016*
Parental education: median (IQR)	14.5 (13–16)	15.3 (12–17.25)	13.5 (12.5–16)	>0.9999	0.8997	0.8735
SANS: median (IQR)	–	6 (6–13)	12 (9–18.75)	–	*0.0435*	
SAPS: median (IQR)	–	2 (0–5)	2 (0.25–7)	–	0.8213	–
BPRS: median (IQR)	–	41 (34–59)	42 (35.5–55.5)	–	0.874	–
WASI: median (IQR)	113 (105–120)	112 (98–118.5)	103 (78–114)	>0.9999	0.6324	0.1448
Antipsychotic dose (CPZ mg: median (IQR)		150 (116.7–300)	133.3 (83.3–225)	–	0.4993	–
Antipsychotic medication: (*n*)	–					
Aripiprazole		2	2			
Chlorpromazine		0	1			
Lurasidone		0	1			
Olanzapine		2	1			
Quetiapine		1	0			
Risperidone		2	4			
More than one atypical		2	0			
Unmedicated		5	2			

*CTL, control; AFF, affective; NON, non-affective; IQR, interquartile range; SANS, Scale for the Assessment of Negative Symptoms; SAPS, Scale for the Assessment of Positive Symptoms; BPRS, Brief Psychiatric Rating Scale; WASI, Wechsler Abbreviated Scale of Intelligence; CPZ, Chlorpromazine equivalent dose.*

### Basal Cytokine and Chemokine Production (Media Alone)

**FIGURE 1:**
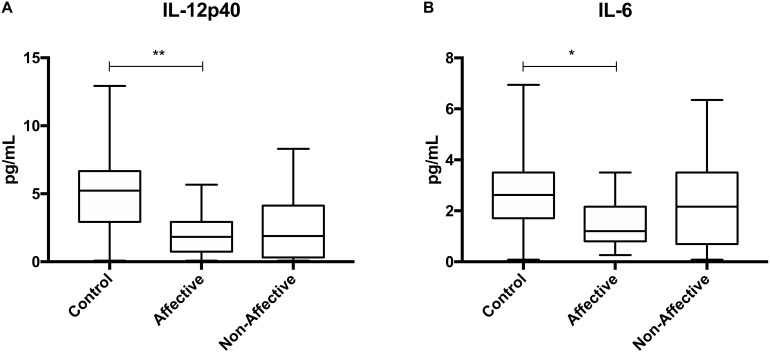
Baseline (media only) cytokine/chemokine levels. Basal macrophage cytokine and chemokine production after culturing for 24 h in RPMI. Concentrations in pg/mL of IL-12p40 **(A)** and IL-6 **(B)** were significantly lower in the AFF group compared to the HC group. Box and whiskers graphs depict median, upper and lower interquartile ranges, *p < 0.05, **p < 0.01. HC *n* = 23, AFF *n* = 14, and NON *n* = 11.

We first assessed baseline macrophage production of inflammatory cytokines, chemokines and growth/anti-inflammatory factors after 24-hour culture in RPMI medium. Kruskal–Wallis tests revealed significant differences between HC, AFF, and NON-groups for production of IL-12p40 (*p* < 0.01) and IL-6 (*p* < 0.05). After correcting for multiple comparisons, Mann–Whitney *U* tests revealed significantly reduced production of inflammatory cytokines IL-12p40 (*p* = 0.0069) and IL-6 (*p* = 0.0191; Table 2, Figure 1) in the AFF group compared to HC. No significant differences were seen when comparing IL-12p70, IL-1β, TNF-α, MCP-1, MIP-1α, MIP-1β, GM-CSF, or IL-10.

**TABLE 2:** Concentration of cytokines/chemokines at baseline (media only).

	HC	AFF	NON	Adjusted *P* values		
	Median	Median	Median	HC vs AFF	AFF vs NON	HC vs NON
	(IQR)	(IQR)	(IQR)			
IL-12p40	5.23	1.835	1.89	*0.0069*	0.8079	***0.0624***
	(2.93–6.67)	(0.7375–2.93)	(0.3125–4.135)			
IL-12p70	2.25	1.765	1.665	0.1689	0.9999	0.3083
	(1.66–2.91)	(0.7425–2.178)	(0.515–2.855)			
IL-1β	0.79	0.81	0.55	0.5525	0.9206	0.7451
	(0.64–1.08)	(0.42–0.97)	(0.29–1.86)			
IL-6	2.62	1.2	2.16	*0.0191*	0.4569	0.4552
	(1.71–3.5)	(0.8–2.16)	(0.7–3.5)			
TNF-α	9.52	6.95	5.775	0.8758	0.3260	0.2780
	(5.19–12.1)	(5.1–23.19)	(2.75–8.943)			
MCP-1	908.7	409.3	548.6	0.1752	0.9362	0.2540
	(413.8–1,663)	(315.9–781.6)	(202.8–1,009)			
MIP-1α	41.12	21.37	12.37	0.1839	0.6939	0.5319
	(5,492–9,912)	(6,281–19,945)	(4,494–14,958)			
MIP-1β	10,189	9,458	7,387	0.9998	0.9999	0.9998
	(9,269–12,945)	(8,377–13,438)	(3,362–10,462)			
GM-CSF	3.74	2.59	3.52	0.8187	0.8187	0.8187
	(2.16–4.7)	(1.58–5.5)	(1.055–4.97)			
IL-10	13.48	9.56	10.96	0.6961	0.8520	0.852
	(8.23–19.51)	(7.56–14.65)	(7.448–26.92)			

*Median and interquartile ranges (IQR) of measured cytokines. Significant P values italicized.Bold indicates P values that were significant prior to multiple correction testing.*

### Cytokine and Chemokine Production After LPS Stimulation

**FIGURE 2:**
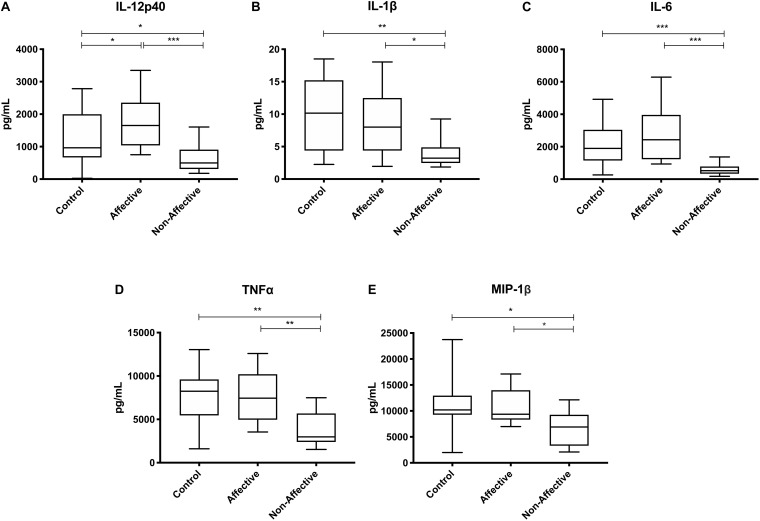
Cytokine/chemokine levels after LPS stimulation. Macrophage cytokine production after culturing for 24 h in RPMI with 10 ng/mL LPS. Macrophages from the NON-group produced decreased concentrations of IL-12p40 **(A)**, IL-1β **(B)**, IL-6 **(C)**, TNF-α **(D)**, and MIP-1β **(E)** compared to the AFF group after stimulation with LPS. NON-macrophages also produced reduced concentrations of IL-12p40 **(A)**, IL-1β **(B)** IL-6 **(C)**, TNF-α **(D)**, and MIP-1β **(E)** compared to HC. Box and whiskers graphs depict median, upper and lower interquartile ranges, *p < 0.05, **p < 0.01, and ***p < 0.001. HC *n* = 23, AFF *n* = 14, and NON *n* = 11.

After stimulating macrophages with LPS for 24 h to measure cytokine production after immune activation, Kruskal–Wallis tests revealed significant differences in IL-12p40 (*p* < 0.01), IL-1β (*p* < 0.01), IL-6 (*p* < 0.001), TNF-α (*p* < 0.01), MIP-1α (*p* < 0.05), and MIP-1β (*p* < 0.05) across the three groups. After correcting for multiple comparisons, Mann–Whitney *U* tests revealed significant decreases in IL-12p40 (*p* = 0.0009), IL-1β (*p* = 0.0248), IL-6 (*p* = 0.0003), TNF-α (*p* = 0.0057), and MIP-1β (p=0.0418) were seen in the NON-group compared to AFF (Table 3, Figure 2). The NON-group also had decreases in IL-12p40 (*p* = 0.0272), IL-1β (*p* = 0.0078), IL-6 (*p* = 0.0008), TNF-α (*p* = 0.0072), and MIP-1β (*p* = 0.0282) compared to HC.

**TABLE 3:** Concentration of cytokines/chemokines after LPS stimulation.

	HC	AFF	NON	Adjusted *P* values		
	Median	Median	Median	HC vs AFF	AFF vs NON	HC vs NON
	(IQR)	(IQR)	(IQR)			
IL-12p40	964	1,652	499.4	*0.0487*	*0.0009*	*0.0272*
	(669.2–1,997)	(1,037–2,356)	(311.8–907.2)			
IL-12p70	70.5	108.5	44.38	0.1304	***0.0527***	0.5155
	(39.4–116.2)	(59.67–228.7)	(32.35–94.13)			
IL-1β	10.16	8.005	3.22	0.5056	*0.0248*	*0.0078*
	(4.395–15.23)	(4.393–12.49)	(2.468–4.885)			
IL-6	1,898	2,430	524	0.3268	*0.0003*	*0.0008*
	(1,147–3,044)	(1,230–3,966)	(336.8– 784.4)			
TNF-α	8,242	7,444	2,982	0.9617	*0.0057*	*0.0072*
	(5,453–9,612)	(4,956–10,200)	(2,393–5,680)			
MCP-1	13,117	9,498	8,489	0.3194	0.5357	0.3033
	(9,294–23,301)	(8,258–17,013)	(4,281–17,646)			
MIP-1α	7,425	16,523	5,374	***0.0837***	***0.0837***	0.6960
	(5,492–9,912)	(6,281–19,945)	(4,494–14,958)			
MIP-1β	10,189	9,458	7,387	0.5535	*0.0418*	*0.0282*
	(9,269–12,945)	(8,377–13,438)	(3,362–10,462)			
GM-CSF	120.1	179.5	114.7	0.3283	0.2332	0.6695
	(107.6–188.7)	(94.59–265.9)	(67.85–171.3)			
IL-10	259.6	199.4	185.4	0.7928	0.7928	0.7928
	(150.7–492)	(130.7–366.3)	(93.95–325.1)			

*Median and interquartile ranges (IQR) of measured cytokines. Significant P values italicized. Bold indicates P values that were significant prior to multiple correction testing.*

### Cytokine and Chemokine Production After LPS Plus IFNγ Stimulation

**FIGURE 3:**
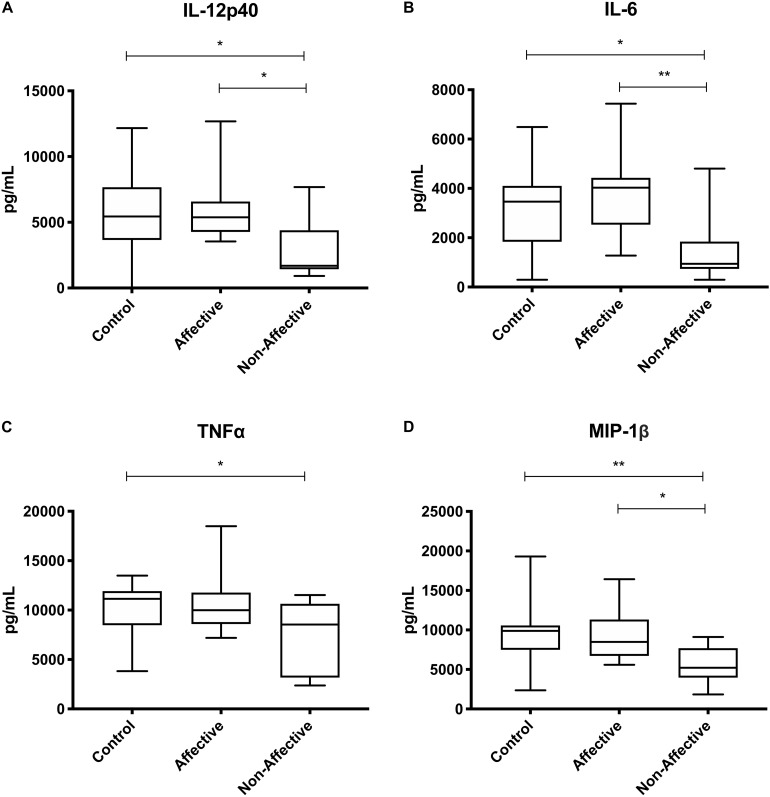
Cytokine/chemokine levels after LPS plus IFNγ stimulation. Macrophage cytokine and chemokine production after culturing for 24 h in RPMI with 10 ng/mL LPS plus 50 ng/ml IFNγ. Macrophages from the NON-group produced decreased concentrations of IL-12p40 **(A)**, IL-6 **(B)**, and MIP-1β **(D)** compared to the AFF group. The NON-group had decreases in IL-12p40 **(A)**, IL-6 **(B)**, TNF-α **(C)**, and MIP-1β **(D)** compared to the HC group after stimulation with LPS plus IFNγ. Box and whiskers graphs depict median, upper, and lower interquartile ranges, *p < 0.05, **p < 0.01. HC *n* = 23, AFF *n* = 14, and NON *n* = 11.

After stimulating with LPS plus IFNγ to induce a classically activated “M1” phenotype, Kruskal–Wallis tests showed significant differences in IL-12p40 (*p* < 0.05), IL-6 (*p* < 0.01), MCP-1 (*p* < 0.05), and MIP-1β (*p* < 0.01) across the three groups. After multiple corrections, Mann–Whitney *U* tests showed significantly decreased production of IL-12p40 (*p* = 0.0143) and IL-6 (*p* = 0.0033) in the NON-group compared to the AFF group (Table 4, Figure 3). IL-12p40 (*p* = 0.0213), IL-6 (*p* = 0.0116), and TNF-α (*p* = 0.0452) were also lower in the NON-group compared to HC. When comparing chemokines, MIP-1β was significantly decreased in the NON-group compared to both the AFF (*p* = 0.0102) and HC (*p* = 0.0021) groups.

**TABLE 4:** Concentration of cytokines/chemokines after LPS plus IFNγ stimulation.

	HC	AFF	NON	Adjusted *P* values		
	Median	Median	Median	HC vs AFF	AFF vs NON	HC vs NON
	(IQR)	(IQR)	(IQR)			
IL-12p40	5,443	5,386	1,690	0.9224	*0.0143*	*0.0213*
	(3,657–7,664)	(4,264–6,587)	(1,425–4,394)			
IL-12p70	848.3	1,195	416	0.2922	***0.1356***	0.2922
	(653–1,253)	(1,017–1,396)	(305.9–1,483)			
IL-1β	13.18	12.96	5.61	0.9324	0.2710	0.2710
	(7.76–16.06)	(7.225–18.72)	(4.11– 13.48)			
IL-6	3,463	4,032	940.6	0.2312	*0.0033*	*0.0116*
	(1,834–4,104)	(2,528–4,429)	(735.6–1,846)			
TNF-α	11,134	9,991	8,542	0.9597	0.1779	*0.0452*
	(8,477–11,926)	(8,597–11,772)	(3,179–10,645)			
MCP-1	14,821	9,101	8,634	***0.0611***	0.8201	***0.0611***
	(9,095–29,783)	(7,607–10,435)	(3,869–11,603)			
MIP-1α	9,277	11,442	6,747	0.4534	0.4201	0.4534
	(5,492–9,912)	(6,281–19,945)	(4,494–14,958)			
MIP-1β	10,189	9,458	7,387	0.6536	*0.0102*	*0.0021*
	(9,269–12,945)	(8,377–13,438)	(3,362–10,462)			
GM-CSF	73.7	94.88	83.1	0.3146	0.3544	0.9450
	(53.77–108.5)	(68.12–227.5)	(53.77–108.4)			
IL-10	117.3	117.7	108.6	0.8677	0.9459	0.9850
	(70.44–148.8)	(73.72–168.1)	(71.37–162.9)			

*Median and interquartile ranges (IQR) of measured cytokines. Significant P values italicized. Bold indicates P values that were significant prior to multiple correction testing.*

### Cytokine and Chemokine Production After LPS Plus IL-4 Stimulation

**FIGURE 4:**
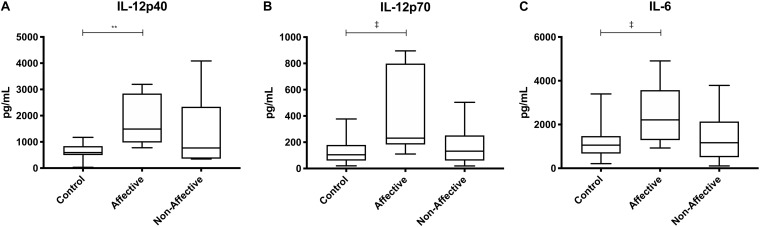
Cytokine/chemokine levels after LPS plus IL-4 stimulation. Macrophage cytokine and chemokine production after culturing for 24 h in RPMI with 10 ng/mL LPS and 40 ng/mL IL-4. Macrophages from the AFF group produced higher concentrations of IL-12p40 **(A)** with trending increases in IL-12p70 **(B)** and IL-6 **(C)** compared to the HC group. Box and whiskers graphs depict median, upper and lower interquartile ranges, **p < 0.01. ^‡^Indicates comparisons that were significant prior to multiple correction testing. HC *n* = 14, AFF *n* = 7, and NON *n* = 6.

After stimulating macrophages with LPS and IL-4 to induce an alternatively activated “M2” phenotype, Kruskal–Wallis tests showed significant differences in IL-12p40 (*p* < 0.01) and IL-12p70 (*p* < 0.05) across the three groups. For pairwise comparisons, Mann–Whitney *U* tests showed significantly increased production of IL-12p40 with increases in IL-12p70 and IL-6 trending to but not reaching statistical significance (Table 5, Figure 4).

**TABLE 5:** Concentration of cytokines/chemokines after LPS + IL-4 stimulation.

	HC *n* = 13	AFF *n* = 9	NON *n* = 5	Adjusted *P* values		
	Median	Median	Median	HC vs AFF	AFF vs NON	HC vs NON
	(IQR)	(IQR)	(IQR)			
IL-12p40	591.7	1,489	767	*0.0024*	0.4137	0.5214
	(499.9–840)	(975.3–2,843)	(360.8–2,338)			
IL-12p70	105	231.6	132.4	***0.0588***	0.1414	0.659
	(60.06–179.5)	(182.6–798.7)	(60.74–252.3)			
IL-1β	6.44	11.52	3.475	0.6494	0.6494	0.6494
	(3.195–12.81)	(3.75–13.33)	(2.548–8.568)			
IL-6	1,056	2,214	1,166	***0.0588***	0.2561	0.968
	(671.3–1,472)	(1,289–3,574)	(509.3–2,137)			
TNF-α	5,874	9,264	6,778	0.6887	0.6922	0.7181
	(3,714–8,727)	(4,123–10,671)	(5,245–7,469)			
MCP-1	9,131	18,951	12,820	0.6378	0.7440	0.7440
	(6,717–15,481)	(6,899–28,525)	(9,090–18,900)			
MIP-1α	6,305	20,000	9,517	***0.1002***	0.5338	0.2282
	(5,492–9,912)	(6,281–19,945)	(4,494–14,958)			
MIP-1β	10,189	9,458	7,387	0.9989	0.9999	0.9989
	(9,269–12,945)	(8,377–13,438)	(3,362–10,462)			
GM-CSF	121.1	174.7	127.3	0.6188	0.8618	0.8618
	(68.34–173.3)	(97.09–386)	(96.98–216.9)			
IL-10	564.7	197.8	271.9	0.9956	0.9956	0.9956
	(277.5–892.8)	(141.6–1,579)	(141–2,406)			

*Median and interquartile ranges (IQR) of measured cytokines. Significant P values italicized. Bold indicates P values that were significant prior to multiple correction testing.*

### Logistic Regression With Receiver Operating Characteristic (ROC) Curve for Discriminating Affective Versus Non-Affective Patients

**FIGURE 5:**
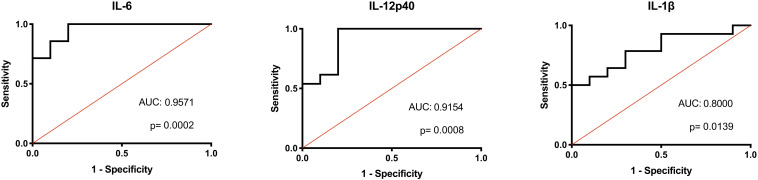
Binary logistic regression with ROC curve output. Binary logistic regression with ROC curve output of the AFF group plotted against those in the NON-group. A receiver operating characteristic (ROC) curve analysis indicated that IL-IL-6, 12p40, and IL-1β levels were significant predictors of affective psychoses (area under ROC curve, 0.9571, 0.9154, and 0.8000, respectively). The diagonal defines random event.

Considering the consistent differences in inflammatory macrophage responses after LPS stimulation between the AFF group compared to the NON-group, we investigated whether these differences had the potential to predict whether psychosis participants would fall into the AFF group or NON-group using binary logistic regression analysis and receiver operating characteristic (ROC) curve output. We first log2 transformed the concentrations of each cytokine to improve goodness of fit (Grund and Sabin, 2010). Unadjusted univariate binary logistic regression analysis of the log2-transformed cytokine concentrations revealed that higher levels of IL-12p40, IL-1β, IL-6, TNF-α, and MIP-1β increased odds of being in the AFF group compared to the NON-group (*p* < 0.05); however, confidence intervals were wide (Supplementary Table S2). ROC analysis indicated that when the log2 concentrations of these cytokines and chemokines were used individually to discriminate between groups, the area under the curve (AUC) ranged from 0.7730 to 0.9571. The most predictive of the cytokines and chemokines measured to discriminate between the AFF and NON-groups were IL-6 and IL-12p40, followed by IL-1β yielding AUC of 0.9571 (*p* = 0.0002), 0.9154 (*p* = 0.0008), and 0.8000 (*p* = 0.0139), respectively (Figure 5).

## Discussion

In order to advance our understanding of innate immune dysfunction in psychotic disorders, our analysis focused on identifying differences in inflammatory cytokines associated with macrophage activation in individuals with either affective or non-affective psychosis. Our approach measured cytokines during a range of stimulatory conditions to identify dysfunction over different possible phenotypes of macrophages, including toward M1 and M2. We found that monocyte-derived macrophages from the NON-group consistently produced lower levels of innate cytokines under inflammatory conditions compared to both HC and AFF. Additionally, the AFF group had lower cytokine production at baseline compared to controls, however, after inflammatory activation, they showed more robust responses when compared to the NON-group. Under conditions of alternative activation, AFF macrophages produced higher amounts of inflammatory cytokines compared to controls. These data support an overall dysfunctional innate immune response both affective and non-affective psychosis groups.

Our findings of decreased responses in the NON-group and increased responses in the AFF group are not completely unexpected. Our results support previous work that looked at gene expression in PBMC and identified an increased M1/decreased M2 signature in BD, but not in SCZ (Brambilla et al., 2014). Two previous studies also identified increased expression of clusters of inflammatory genes in monocytes from affective and psychotic patients, with some overlap but also some distinct differences between BD, SCZ, and MDD. All groups showed increases in inflammatory gene expression however the SCZ group had downregulation of transcription factors for adhesion and chemotactic molecules (Drexhage et al., 2010b; Carvalho et al., 2014). It is important to note that these cells were not stimulated, so their results only provide insight as to their behavior at rest. A recent study by Ascoli et al. (2019) found that inflammatory macrophage responses after M1/M2 skewing diminished with disease progression in BD, where “early stage” BD macrophages more closely resembled HC macrophage responses, but in later-stage disease the responses were attenuated. Our patients were not considered chronically ill, all were within a year of first symptoms and our findings that macrophages in the AFF group respond similarly to HC after LPS and M1 stimulation supports this research.

Chronic activation of macrophages has been a proposed etiological mechanism of SCZ for decades (Smith and Maes, 1995), and many studies have indicated circulating pro-inflammatory cytokines associated with these cells are increased in SCZ, BD, and MDD (Dowlati et al., 2010; Modabbernia et al., 2013; Upthegrove et al., 2014; Pillinger et al., 2018), Under certain circumstances such as with LPS stimulation, when macrophages are previously exposed to LPS they can become refractory to this stimuli. This could be the mechanism behind the decreased macrophage activity seen in the NON-group (Biswas and Lopez-Collazo, 2009). This does not explain responses similar to HC after LPS and M1 stimulation in the AFF group, however, some cytokine studies have proposed that inflammation is not consistently present in AFF disorders, rather they vary depending on state of disorder (Munkholm et al., 2013; Goldsmith et al., 2016), therefore it is possible that AFF cells are not experiencing the same levels of chronic activation as seen in SCZ. These differential responses suggest that a different type of immune dysfunction may be involved in AFF disorders.

The increased inflammatory responses in the AFF macrophages after alternative activation was not expected. Production of IL-6 and IL-12 is generally associated with classically activated macrophages. In the presence of IL-4, macrophages typically skew to the M2 phenotype; however, it is important to note there is high heterogeneity in M2 macrophages and the specific phenotype driven by IL-4 exposure is M2a, commonly seen activated in T-helper cell type 2 (Th2) parasite and allergic responses (Luzina et al., 2012). The atypical response to IL-4 in the AFF macrophages may provide a clue that there could be different types of immune dysfunction involved in affective versus non-affective psychotic disorders. For example, cytokine meta-analyses show elevated IL-4 in BD but decreased IL-4 in FEP and SCZ (Modabbernia et al., 2013; Goldsmith et al., 2016). Increased IL-4 mRNA was also previously identified in BP patients compared to SCZ (Brambilla et al., 2014), and affective disorders may accompany a higher risk of allergic diseases than psychotic disorders (Tzeng et al., 2018). We recently measured plasma cytokines in a cohort of FEP patients and found elevated plasma IL-2, IL-6, and IFNγ in SCZ patients, however, only IL-10 was elevated in BD compared to healthy controls (Lesh et al., 2018). These differential findings when comparing disorders could potentially be useful for distinguishing between AFF and NON FEP patients early in the diagnostic process, similar to the predictive ROC curves we provided.

Cytokines are critical mediators of the immune response, and often function as messengers to elicit inflammatory or anti-inflammatory responses from other cells (Turner et al., 2014). These cytokines are tightly regulated within the central nervous system (CNS), as appropriate amounts are necessary for neurodevelopment, homeostasis, synaptic pruning and plasticity (Deverman and Patterson, 2009). Circulating inflammatory cytokines such as IL-1β, IL-6, and TNF-α are able to cross the blood-brain-barrier, and are known to influence behavior, therefore they have the capacity to directly influence the activity of resident immune cells such as the microglia (Yarlagadda et al., 2009). Although not technically the same cells due to differing ontogeny, the yolk-sac derived microglia are myeloid cells that have similar gene expression and cytokine production profiles as monocytes and macrophages (Takahashi et al., 2016). Microglia play a major role in neurogenesis, and are responsible for eliminating excess neuronal precursors in the cerebral cortex during this process (Cunningham et al., 2013). Additionally, they are crucial for the maintenance of synapses by constantly surveilling and interacting with all regions of the neuron including dendritic processes, which they refine through pruning (Paolicelli et al., 2011), and failure of this process leads to impaired connectivity and altered behavior in mice (Zhan et al., 2014). Perivascular and meningeal macrophages (also yolk-sac derived) as well as choroid plexus macrophages (which can be replenished from circulating monocytes) also have the potential to skew to an M1 phenotype when inflammatory cytokines are present in the milieu (Goldmann et al., 2016; Prinz et al., 2017). During neuroinflammatory events, it is possible for infiltrating monocytes to enter the CNS (Minogue, 2017), further contributing to neuroinflammation.

Dysfunctional activation of microglia and neuroinflammation have been proposed to contribute to pathological mechanisms in psychiatric disorders, and these can occur under conditions of systemic inflammation and macrophage activation (Perry et al., 2007; Takahashi et al., 2016). Considering the important role that microglia play in neuronal survival and maintenance of synapses, it has been suggested that failure of this maintenance or dysregulation of microglia may play a role in cortical gray matter thinning during aging (Vidal-Pineiro et al., 2020). Excess cortical thinning is common pathology seen in psychiatric disorders, for example, meta-analyses of voxel-based morphometry to measure gray matter volume found that the dorsal anterior cingulate, right insula, and left insula gray matter loss are consistently seen across a diverse group of psychiatric conditions that included SCZ, BD and depression, however, MDD patients had greater gray matter losses in the hippocampus and amygdala while gray matter increases were seen exclusively in the striatum of SCZ spectrum patients (Goodkind et al., 2015). In a previous study identifying differences in plasma cytokines in SCZ and BD, researchers found an inverse relationship between inflammatory cytokines and gray matter thickness in SCZ, but not in BD (Lesh et al., 2018). Antipsychotic medication has been associated with gray matter loss, however, one study found progressive loss of cortical thickness despite no use of antipsychotic drugs in subjects at high-risk for developing a psychotic disorder who later converted to psychosis, suggesting that this phenomenon is part of the pathophysiology of these disorders (Cannon et al., 2015). Differences in microglial phenotype and function could be contributing to these differences, and investigation of post mortem brain tissue as well as *in vivo* positron emission tomography (PET) studies have provided some evidence of microglia activation in these disorders (Bayer et al., 1999; van Berckel et al., 2008; Doorduin et al., 2009; Dean et al., 2010; Rao et al., 2010; Fillman et al., 2013; Haarman et al., 2014; Setiawan et al., 2015; Bloomfield et al., 2016; Trepanier et al., 2016; Holmes et al., 2018; Richards et al., 2018; Setiawan et al., 2018). However, findings have not always been consistent and complexity due to variations in methodology complicate the overall picture of neuroinflammation in SCZ, BD, and MDD. A theme that has emerged during investigation of neuroinflammation is that increases may be associated with the state of disease, contributing to variations in findings (reviewed in Hughes and Ashwood, 2020).

There are several limitations to our study that must be taken into account when interpreting results. Our sample sizes for both the AFF and NON-groups were relatively small, and this was most apparent during regression analysis which suggested that higher concentrations of inflammatory cytokines after LPS stimulation are associated with the AFF macrophages. However, the wide confidence intervals indicate these data need to be replicated in larger cohorts for confirmation of this analysis. Moreover, patients were assessed within one year of diagnosis, not necessarily during active first-episode, and therefore may have variability in inflammatory and behavioral status based on the possibility that the state of disease may influence level of immune activation. Many of the psychotic subjects were taking medications including anti-psychotics which have been shown to alter cytokine production in psychotic patients (Noto et al., 2015), however, these findings are not consistent across studies (Theodoropoulou et al., 2001). Despite these limitations, our study had several advantages over previous cytokine studies in neuropsychiatric disorders. It is important to note that circulating cytokines can have significant individual variation, and simple measurement of plasma cytokines does not provide information about the type of immune cell(s) producing the cytokines. By differentiating circulating monocytes into macrophages, we are able to identify dysfunction specific to this cell set and therefore provide functional information about how macrophages might be behaving during various states of immune activation. Additionally, by culturing and differentiating monocytes into macrophages over a seven-day period prior to stimulation, we likely minimized the effect of any medications on the behavior of these cells.

## Conclusion

It is clear from an abundance of research that immune dysfunction is present and likely plays a role in neuropsychiatric disorders. In summary, we report differential monocyte dysfunction in psychotic disorders with and without a mood component. Our work expands on previous studies that identified differences and similarities in macrophage responses across SCZ, BD and MDD, and suggest that affective psychoses may have a different type of immune dysfunction than primary psychotic disorders, which may involve elevated IL-4 and dysfunctional M1/M2 skewing. It is possible other circulating cell types may be responsible for the chronic inflammatory findings in these disorders. Future studies could attempt to screen other circulating immune cells for similar differential patterns, or characterize macrophages further through flow cytometric analysis and transcriptomics. This could help identify if there are differences across groups in ability of macrophages to differentiate, which may be contributing to the differences in cytokine production seen in our study. By better characterizing immune dysfunction in these disorders we can identify targets for therapeutics that might be beneficial for affective and non-affective psychotic disorders.

## Data Availability Statement

The raw data supporting the conclusions of this article will be made available by the authors, without undue reservation.

## Ethics Statement

The studies involving human participants were reviewed and approved by University of California, Davis Institutional Review Board. Written informed consent to participate in this study was provided by the participants’ legal guardian/next of kin.

## Author Contributions

HH and EM-K contributed to the design of the study, performed the data analyses, contributed to the data interpretation, and drafting of the manuscript. HH, HY, and EM-K processed the blood, and ran the assays on the macrophage cultured samples. TL and CC contributed to the interpretation of the data, and revisions of the manuscript. PA designed the study, and made substantial contributions to interpretation of the data, and drafting and revisions of the manuscript. All authors read and approved the final manuscript.

## Conflict of Interest

The authors declare that the research was conducted in the absence of any commercial or financial relationships that could be construed as a potential conflict of interest.

## Supplementary Material

Supplementary Figure S1 Macrophage cytokine and chemokine production after culturing for 24 h under four conditions: RPMI alone, RPMI with 10 ng/mL LPS, RPMI with 10 ng/mL LPS plus 50 ng/ml IFNγ, or RPMI with 10 ng/mL LPS and 40 ng/mL IL-4. Box and whiskers graphs depict median, upper, and lower interquartile ranges, ^∗^*p* < 0.05, ^∗∗^*p* < 0.01, and ^∗∗∗^*p* < 0.001. ^‡^Indicates pairwise comparisons that were significant prior to multiple correction testing.
