# Supplementary figures and images for: Differential Macrophage Responses in Affective Versus Non-Affective First-Episode Psychosis Patients
Source: Front Cell Neurosci. 2021 Feb 24;15:583351. doi: 10.3389/fncel.2021.583351 (PMC7943877; doi:10.3389/fncel.2021.583351)

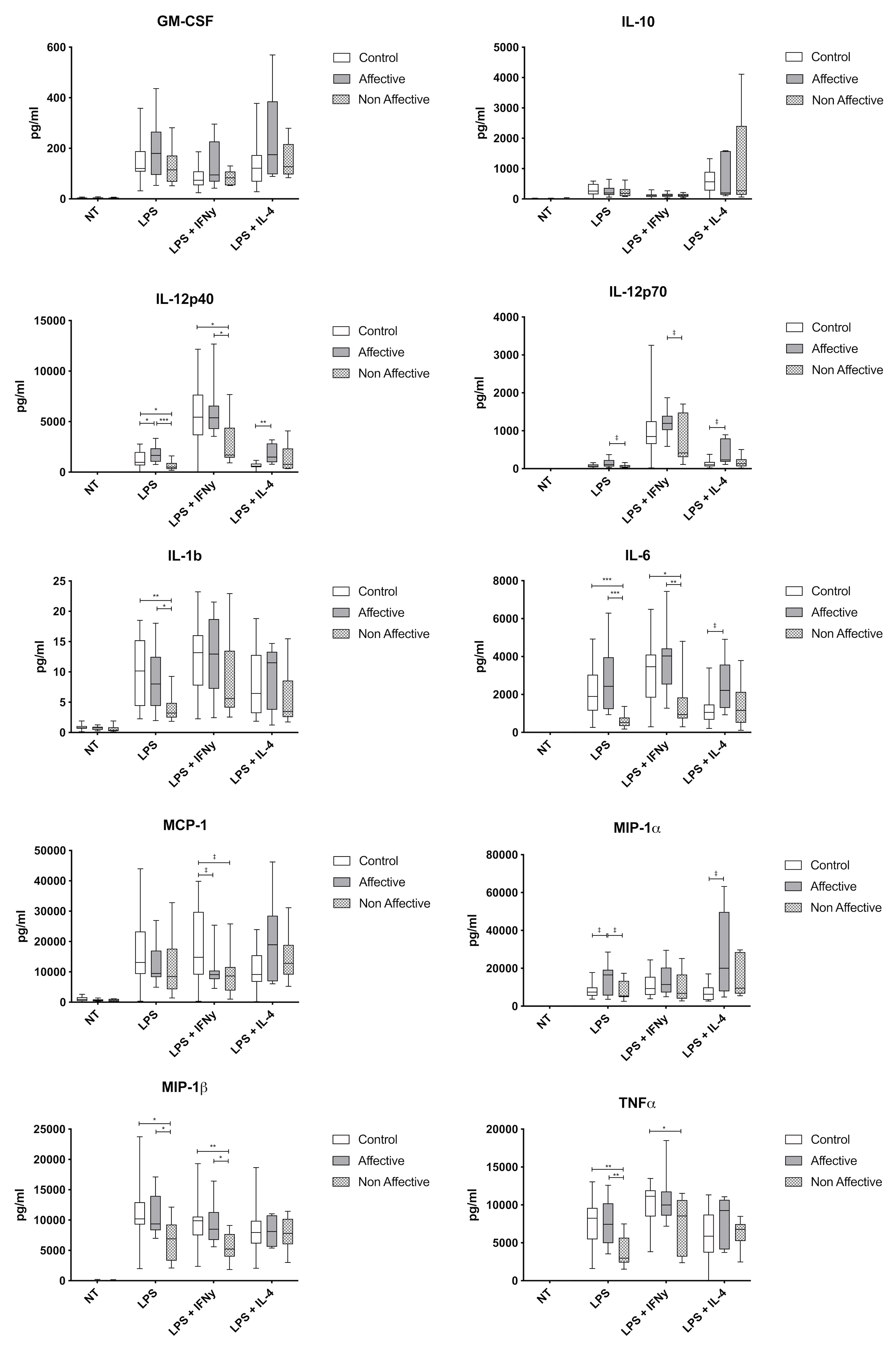

Supplement: Supplementary Figure S1 — Macrophage cytokine and chemokine production after culturing for 24 h under four conditions: RPMI alone, RPMI with 10 ng/mL LPS, RPMI with 10 ng/mL LPS plus 50 ng/ml IFNγ, or RPMI with 10 ng/mL LPS and 40 ng/mL IL-4. Box and whiskers graphs depict median, upper, and lower interquartile ranges, ∗p < 0.05, ∗∗p < 0.01, and ∗∗∗p < 0.001. ‡Indicates pairwise comparisons that were significant prior to multiple correction testing. [file Image_1.JPEG]
