# Supplementary material for: Differential Macrophage Responses in Affective Versus Non-Affective First-Episode Psychosis Patients
Source: Front Cell Neurosci. 2021 Feb 24;15:583351. doi: 10.3389/fncel.2021.583351 (PMC7943877; doi:10.3389/fncel.2021.583351)
Supplement: Supplementary file 2 [file Table_1.docx]

| Supplemental Table 1: ANCOVA with CPZ dosage as covariate | | | |
| --- | --- | --- | --- |
|  |  |  |  |
| LPS Stim | F | P value | R squared |
| IL-12 p40 | F(1,20)=21.10 | p<0.001 | 0.514 |
| IL-1β | F(1,21)=31.82 | p=0.011 | 0.273 |
| IL-6 | F(1,21)=14.28 | p<0.001 | 0.625 |
| TNF-α | F(1,21)=7.82 | p=0.001 | 0.417 |
| MIP-1β | F(1,22)=6.84 | p=0.016 | 0.281 |
|  |  |  |  |
|  |  |  |  |
| LPS + IFNγ Stim | F | P value | R squared |
| IL-12 p40 | F(1,21)=12.77 | p=0.002 | 0.446 |
| IL-6 | F(1,22)=16.90 | p<0.001 | 0.466 |
| MIP-1β | F(1,22)=10.16 | p=0.004 | 0.321 |
