# Supplementary material for: Differential Macrophage Responses in Affective Versus Non-Affective First-Episode Psychosis Patients
Source: Front Cell Neurosci. 2021 Feb 24;15:583351. doi: 10.3389/fncel.2021.583351 (PMC7943877; doi:10.3389/fncel.2021.583351)
Supplement: Supplementary file 3 [file Table_2.DOCX]

Supplemental Table 2: SPSS binary logistic regression output


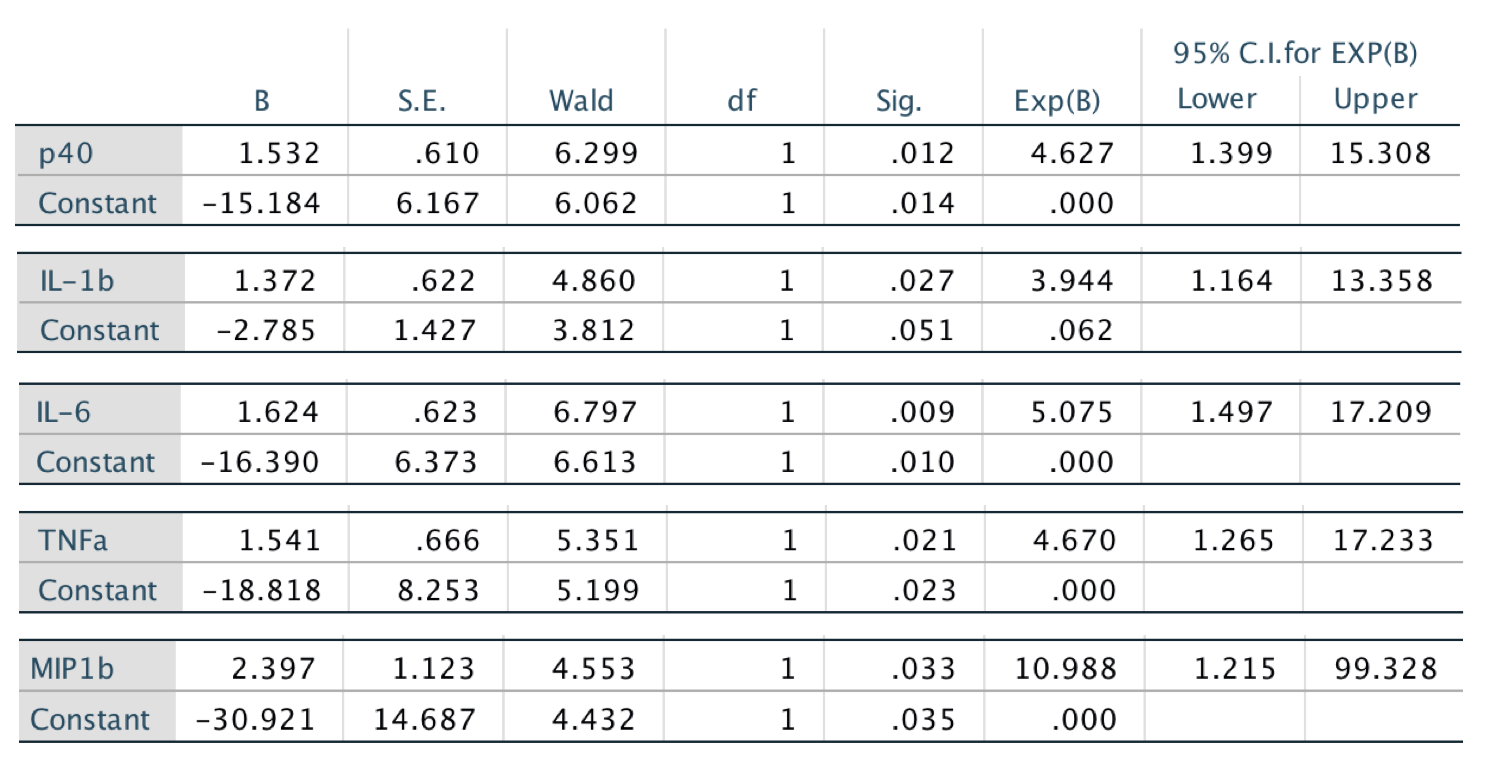


Regression coefficient (B), standard error (S.E.), Wald statistic, degrees of freedom (df), significance (sig.) odds ratio (ExpB), confidence interval (C.I.)
